# Prognostic value of preoperative myocardial fibrosis assessment by delayed enhancement cardiac magnetic resonance imaging in patients undergoing mitral valve repair

**DOI:** 10.1186/1532-429X-16-S1-P284

**Published:** 2014-01-16

**Authors:** Kongkiat Chaikriangkrai, Juan C Lopez-Mattei, Homam Ibrahim, Stephen H Little, William Zoghbi, Miguel A Quinones, Gerald Lawrie, Dipan J Shah

**Affiliations:** 1 Medicine, The Methodist Hospital, Houston, Texas, USA; 2 Methodist DeBakey Heart & Vascular Center, The Methodist Hospital, Houston, Texas, USA; 3 Cardiothoracic surgery, The Methodist Hospital, Houston, Texas, USA

## Background

Cardiac MRI has been shown to be the gold standard for noninvasive assessment of myocardial fibrosis. The extent of fibrosis has been associated with worse outcomes in patients undergoing CABG surgery. The presence of fibrosis and associated clinical features of patients with chronic mitral regurgitation undergoing mitral valve repair have not been described. The objective of this study is to examine prognostic value of preoperative myocardial fibrosis assessment by cardiac MRI in patients with chronic mitral regurgitation undergoing mitral valve repair.

## Methods

This is a prospectively enrolled consecutive series of 48 subjects with chronic mitral regurgitation who underwent cardiac MRI followed by mitral valve repair using the non-resectional dynamic technique from August 2008 - April 2013. Pre-operative fibrosis burden, other cardiac MRI and clinical variables were collected. Differences among variables between subjects with and without fibrosis were statistically calculated.

## Results

The cohort had a mean (SD) age of 61(13) years and included 33 men (69%). Median postoperative follow up interval was 53.5 days (Interquartile range 10 and 442 days). Mean (SD) interval between cardiac MRI and mitral valve repair was 19.58 (18.37) days. Fibrosis was present in 19 subjects (39.6%) with a median fibrosis burden of 4% of the left ventricle. Within the fibrosis group, 9 (47.4%) had a non-coronary artery disease fibrosis pattern. Comparison of selected baseline clinical and cardiac MRI variables is shown in the Table 1. Postoperative clinical outcomes are shown in the Table 2. In multivariable analysis, presence of preoperative fibrosis is an independent predictor for postoperative cardiac pacing (OR 7.652; 95%CI 1.636, 35.783, p0.010), postoperative significant arrhythmia (OR 4.403; 95%CI 0.997, 19.443, p0.050) and a composite of ICU readmission/postoperative significant arrhythmia/postoperative cardiac pacing (OR 5.057; 95%CI 1.130, 22.622, p0.034).

**Table 1:** Baseline clinical and cardiac MRI characteristics categorized by presence of myocardial fibrosis

Variables	All patients (N = 48)	NO fibrosis (N = 29)	WITH fibrosis (N = 19)	P
Age, years (SD)	61 (13)	59 (11)	65 (15)	0.112

Male sex, No. (%)	33 (69)	18 (62.1)	15 (78.9)	0.217

BMI, kg/m2 (range)	28.4 (19.0-56.1)	27.0 (19.0-56.1)	29.4 (19.5-53.5)	0.348

Functional mitral regurgitation, No. (%)	13 (27.1)	5 (17.2)	8 (42.1)	0.06

History of diabetes mellitus, No. (%)	7 (14.6)	3 (10.3)	4 (21.1)	0.31

History of dyslipidemia, No. (%)	23 (47.9)	11 (37.9)	12 (63.2)	0.09

History of hypertension, No. (%)	30 (62.5)	16 (55.2)	14 (73.7)	0.20

Hemoglobin level on admission, g/dL (range)	13 (7-16)	13.0 (8.0-15.0)	13.0 (7.0-16.0)	0.574

Statins, No. (%)	14 (29.2)	6 (20.7)	8 (42.1)	0.110

ACEI or ARB, No. (%)	19 (39.6)	8 (27.6)	11 (57.9)	0.036

β blockers, No. (%)	25 (52.1)	12 (41.4)	13 (68.4)	0.067

Aspirin, No. (%)	22 (45.8)	13 (44.8)	9 (47.4)	0.863

Cardiac MRI LVEF, % (SD)	63.04 (11.86)	63.31 (12.34)	62.63 (11.40)	0.85

Cardiac MRI RVEF, % (SD)	52.00 (10.33)	52.97 (10.62)	48.84 (9.60)	0.18

Cardiac MRI LVEDV, mL (SD)	200.00(61.34)	199.42 (57.56)	198.34 (68.33)	0.95

Cardiac MRI LVESV, mL (SD)	68.50 (41.03)	75.63 (40.25)	76.50 (43.31)	0.94

**Table 2:** Postoperative clinical outcomes categorized by presence of myocardial fibrosis

Clinical outcomes	All patients (N = 48)	NO fibrosis (N = 29)	WITH fibrosis (N = 19)	P
Significant arrhythmia*, No. (%)	24 (50.0)	10 (34.5)	14 (73.7)	0.008

Cardiac pacing, No. (%)	20 (41.7)	7 (24.1)	13 (68.4)	0.002

ICU readmission or significant arrhythmia or cardiac pacing	25 (52.1)	4 (17.4)	15 (60.0)	0.003

* Atrial fibrillation, sinus bradycardia requiring cardiac pacing, AV blocks requiring cardiac pacing

## Conclusions

Presence of preoperative myocardial fibrosis derived from cardiac MRI is associated with worse postoperative outcomes in patients with chronic mitral regurgitation undergoing mitral valve repair.

## Funding

None.

